# Optimization of DRASTIC method by artificial neural network, nitrate vulnerability index, and composite DRASTIC models to assess groundwater vulnerability for unconfined aquifer of Shiraz Plain, Iran

**DOI:** 10.1186/s40201-016-0254-y

**Published:** 2016-08-09

**Authors:** Mohammad Ali Baghapour, Amir Fadaei Nobandegani, Nasser Talebbeydokhti, Somayeh Bagherzadeh, Ata Allah Nadiri, Maryam Gharekhani, Nima Chitsazan

**Affiliations:** 1 Department of Environmental Health Engineering, School of Health, Shiraz University of Medical Sciences, Shiraz, IR Iran; 2 Department of Civil Engineering, College of Engineering, Shiraz University, Shiraz, IR Iran; 3 Department of Hydrogeology, Ab Ati Pazhooh Consulting Engineers, Shiraz, IR Iran; 4 Department of Earth Science, Faculty of Science, University of Tabriz, Tabriz, East Azarbaijan, IR Iran; 5 Research Engineer at EnTech Engineering, PC11 broadway 21st floor, New York, NY 10004 USA

**Keywords:** Composite DRASTIC, Nitrate vulnerability, Artificial neural network, Shiraz aquifer

## Abstract

**Background:**

Extensive human activities and unplanned land uses have put groundwater resources of Shiraz plain at a high risk of nitrate pollution, causing several environmental and human health issues. To address these issues, water resources managers utilize groundwater vulnerability assessment and determination of protection. This study aimed to prepare the vulnerability maps of Shiraz aquifer by using Composite DRASTIC index, Nitrate Vulnerability index, and artificial neural network and also to compare their efficiency.

**Methods:**

The parameters of the indexes that were employed in this study are: depth to water table, net recharge, aquifer media, soil media, topography, impact of the vadose zone, hydraulic conductivity, and land use. These parameters were rated, weighted, and integrated using GIS, and then, used to develop the risk maps of Shiraz aquifer.

**Results:**

The results indicated that the southeastern part of the aquifer was at the highest potential risk. Given the distribution of groundwater nitrate concentrations from the wells in the underlying aquifer, the artificial neural network model offered greater accuracy compared to the other two indexes. The study concluded that the artificial neural network model is an effective model to improve the DRASTIC index and provides a confident estimate of the pollution risk.

**Conclusions:**

As intensive agricultural activities are the dominant land use and water table is shallow in the vulnerable zones, optimized irrigation techniques and a lower rate of fertilizers are suggested. The findings of our study could be used as a scientific basis in future for sustainable groundwater management in Shiraz plain.

## Background

Shiraz plain located in southwest of Iran is highly dependent on groundwater for its economic and demographic development. However, urbanization and agricultural activities have caused groundwater contamination by several types of pollutants such as nitrate. Presence of nitrate in the water resources can pose health risks to humans. Therefore, water resources managers are concerned about health and ecological effects of water contaminated with nitrate [1, 2]. Nitrated water can cause blue baby syndrome and certain types of cancer, including cancer of digestive system, stomach, colon, bladder, ovaries, and testicles [3]. Therefore, assessment of groundwater vulnerability to detect the vulnerable areas of aquifers is very important in order to manage groundwater resources.

The concept of aquifer vulnerability was first introduced by Marget. This concept refers to the sensitivity of an aquifer to deterioration due to an external action and is based on the assumption that physical environment may provide some degrees of protection to groundwater against contaminants entering the subsurface zone. Consequently, some land areas are more vulnerable to groundwater contamination than others [4, 5].

There are two main types of vulnerability assessment: intrinsic vulnerability and specific vulnerability. The first term refers to the intrinsic property of groundwater system to human or natural impacts. The most leading model of the intrinsic vulnerability is DRASTIC index. DRASTIC index was introduced by United States Environmental Protection Agency (USEPA) for the first time [6, 7]. It is an abbreviation for seven main parameters in hydro-geological system, which control groundwater contamination. These parameters are Depth to water table (D), net Recharge (R), Aquifer media (A), Soil media (S), Topography (T), Impact of the vadose zone (I), and hydraulic Conductivity (C). In contrast, specific vulnerability is defined as the risk of pollution due to the potential impact of land uses. Based on this definition, it seems that model used in the specific vulnerability are more appropriate for forecasting groundwater vulnerability to nitrate pollution [8]. The Composite DRASTIC is the most widespread method of evaluation of the specific vulnerability. The CD model for the first time was proposed by Secunda et al [9]. In this model, in order to evaluate the potential risk of groundwater nitrate pollution, land use parameter is added to seven main hydrogeological parameters of DRASTIC index, then they were integrated through an additive formulation to estimate the specific vulnerability of a certain area. This model was successfully applied in Sharon region of Israel [9], Azraq basin of Jordan [10] and Hajeb-Jelma aquifer of Tunisia [11]. However, it was claimed that the additive formulation might fail to reflect the protective effect of land uses that do not have any adverse effects on groundwater quality [12]. Thus, a new approach based on a multiplicative model and focused on nitrate pollution has been proposed which is called Nitrate Vulnerability (NV) index. For the first time, Martinez-Bastida [12] suggested to utilize the NV index for assessing the risk of nitrate pollution in Central Spain. He declared using the NV index results in greater accuracy in estimations of specific vulnerability and designation of nitrate vulnerable zones in comparison to the CD index.

Both NV and DC methods rely to expert for assigning weights and rates of the parameters. Recently artificial intelligence (AI) models including artificial neural networks successfully utilized to decrease subjectivity in assessment of groundwater vulnerability [13–15]. ANN is a universal approximator to surrogate complex systems [16]. The most commonly used neural network is the multi-layer perceptron (MLP) with supervised training method that consists of one input layer, hidden layers, and one output layer [17, 18]. To apply ANN to the DRASTIC index, there are seven neurons in the input layer corresponding to the input data (D,R,A,S,T,I, and C), four neurons in the hidden layer, and one neuron in the output layer.

There is an ongoing discussion in the literature regarding the performance of different models in generating the specific vulnerability maps. This study aims to clarify the issue by comparing the results of CD, NV and ANN models for specific vulnerability assessment. The study uses Shiraz unconfined aquifer as a real world example for conducting this comparison.

## Methods

### The study area

**Fig. 1:**
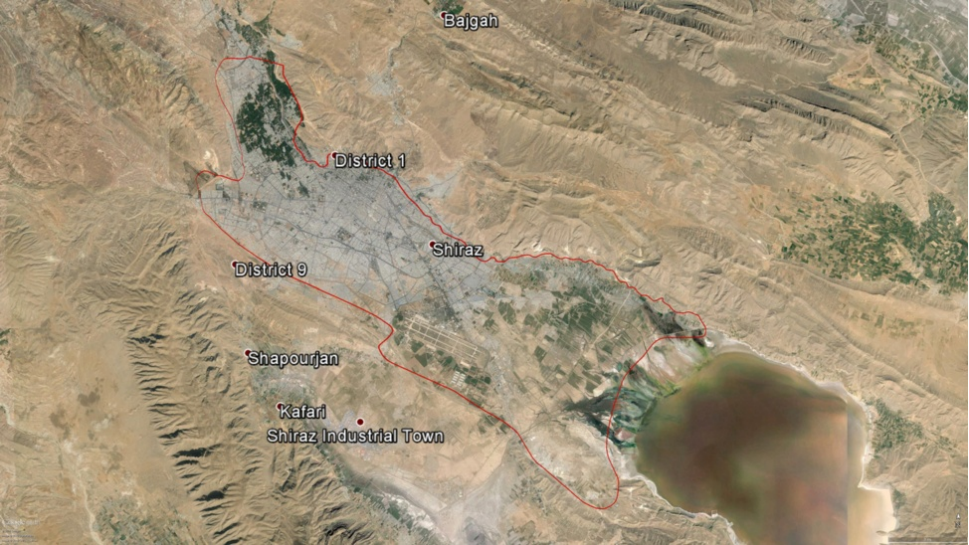
Satellite image of Shiraz plain

The study area was Shiraz plain located in Fars province, Iran (Fig. 1), which is a part of Maharlu lake catchment. The Shiraz plain area is approximately 300 km^2^ and it lies between longitudes 52^0^ 29^′^ and 52^0^ 36^′^ E and altitudes 29^0^ 33^′^ and 29^0^ 36^′^ N. From the north and northwest, the Shirza Plain, is limited by the Baba Koohi Kaftrak mountain heights and Drak Mountain, respectively. From the south, the Shiraz Plain extends to the Maharlu Lake.

Studies have shown that Shiraz plain is an alluvial aquifer with sequences of sand and clay, where the groundwater exists in the sand layers. Alluvial deposits are a sequence of sand and clay/silt layers with various thicknesses.

In addition, geophysical explorations in the plain have demonstrated that Shiraz plain's water-bearing layer in greater depths, it suffers from inappropriate quality. Previous studies indicated that the Shiraz Plain groundwater system is consist of a surface unconfined aquifer and a deep aquifer. Shallow groundwater goes down to a water table to the depth of 40 m, while deep groundwater ranges from about 40 m of depth to 200 m [19]. In the present study, three different models; i.e., CD and NV and ANN models, were used for assessment of vulnerability and determination of groundwater protection zones of Shiraz plain unconfined (shallow) aquifer.

Two seasonal rivers of Khoshkrud and Chenarrahdar exist in Shiraz area. Chenarrahdar River comes from Rahdar bridge area into Shiraz and goes through the southern angle of the city and after absorbing the surface runoff, it joins Maharlu Lake.

The seasonal Khoshkrud River starts from Golestan and Ghalat mountains heights and after joining Khoshk River, it enters the Shiraz plain. Agricultural runoff and industrial wastewater enter this river at the south eastern part of Shiraz city and it finally joins Maharlu Lake. With respect to geology, the formations that outcrop in the study area, from old to new, are: Tarbor formation (Campanian to Maastrichtian), Pabdeh–Gurpi formation (Paleocene), Sachun formation (Paleocene), Jahrom formation (Eocene), Asmari formation (Oligocene), Razak formation (L. Miocene), Agha Jari formation (U. Miocene to L. Pliocene), Bakhtyari formation (U. Pliocene to L. Pleistocen), and quarternary alluvial deposits. The Asmari limestone formation has the most outcrops in the study area. The Shiraz Plain is located in a semi-arid climate zone with the average height of 1540 (m), annual mean precipitation of 365.3 (mm) and average temperature of 18.04 °C.

### The Composite DRASTIC index (additive model)

CD index is an adaption of the DRASTIC index with the addition of a new parameter (L) to define the risk associated with land use (L). The objective of this approach is to evaluate the potential effect of extensive land use on groundwater quality resulting from alteration of the soil matrix and unsaturated zone media over time.

The DRASTIC Index takes into account seven parameters of the geological and hydrological environments, namely depth to water table (D), net Recharge (R), Aquifer media (A), Soil media (S), Topography (T), Impact of the vadose zone (I), and hydraulic Conductivity (C).

According to the effects of parameters on the probable vulnerability, a relative numerical weight from 1 to 5 is given to each parameter, with numbers 1 and 5 representing the least and the most effective, respectively. In addition, these seven parameters are divided into ranges and then receive a number from 1 to 10 according their influence on vulnerability. At the end, after collecting and digitizing the hydro-geological information using GIS, in order to prepare vulnerability maps, the information is overlaid and integrated and the result is a new layer called DRASTIC index (equation 1).

1 $$ \mathrm{DRASTIC}\kern0.5em \mathrm{index}=Dr\kern0.5em Dw\kern0.5em +Rr\kern0.5em Rw+Ar\kern0.5em  Aw+Sr\kern0.5em Sw+Tr\kern0.5em Tw+Ir\kern0.5em Iw+Cr\kern0.5em Cw $$

In this equation: D, R, A, S, T, I, and C are the abbreviations of the seven effective hydro-geological parameters. Besides, subscripts “r” and “w” represent the corresponding ratings and weights in Table 1 [20–22].

**Table 1:** Ratings and weights given to the DRASTIC parameters [6]

Depth to water table (m)		Topography slope (%)		Hydraulic conductivity (m day -1)	
Range	Rating	Range	Rating	Range	Rating
0.0–1.5	10	0–2	10	0–4.1	1
1.5–4.6	9	0–2	9	4.1–12.2	2
4.6–9.1	7	6–12	5	12.2–28.5	4
9.1–15.2	5	12–18	3	28.5–40.7	6
15.2–22.9	3	>18	1	40.7–81.5	8
22.9–30.5	2				
>30.5	1				
Soil media		Aquifer media		Impact of the vadose zone	
Range	Rating	Range	Rating ^a^	Range	Rating ^a^
Thin or absent	10	Massive shale	1–3 (2)	Confining layer	1
Gravel	10	Metamorphic/igneous	2–5 (3)	Silt/clay	2–6 (3)
Sand	9	Weathered metamorphic/igneous	3–5 (4)	Shale	2–6 (3)
Peat	8	Glacial till	4–6 (5)	Limestone	2–5 (3)
Shiniking and/or aggregated clay	7	Bedded sandstone, limestone and shale sequence	5–9 (6)	Sandstone	2–7 (6)
Loam	5	Massive sandstone	4–9 (6)	Bedded limestone, sandstone and shale	4–8 (6)
Silty loam	4	Massive limestone	4–9 (8)	Sand and gravel with significant silt and clay	4–8 (6)
Clay loam	3	Sand and gravel	4–9 (8)	Sand and gravel	4–8 (8)
Muck	2	Basalt	2–10 (9)	Basalt	2–10 (9)
Non-shrinking and non-aggregated clay	1	Karst limestone	9–10 (10)	Karst limestone	8–10 (10)
Parameters	Relative weight				
Depth to water table	5				
Impact of the vadose zones	5				
Net recharge	4				
Aquifer media	3				
Hydraulic conductivity	3				
Soil media	2				
Topography slop	1				

^a^Typical rating in parentheses according to Aller et al. [6]

To determine Composite DRASTIC index, an additional parameter is added to DRASTIC index, which is land use. Therefore, the CD index is calculated using the following equation:

2 $$ CD\kern0.5em  index=Dr\kern0.5em Dw+Rr\kern0.5em Rw+Ar\kern0.5em  Aw+Sr\kern0.5em Sw+Tr\kern0.5em Tw+Ir\kern0.5em Iw+Cr\kern0.5em Cw+Lr\kern0.5em Lw $$

Where L_r_ is the rating of the potential risk associated with land use, L_w_ is the relative weight of the potential risk associated with land use (according to Table 2) and the rest of the parameters are the same as equation 1.

**Table 2:** Ranges and ratings applied to the potential risk associated with land use (L) according to the CD index [9]

Land use category^a^	Lr
Urban areas	8
Irrigated field crops	8
Orchards	6
Uncultivated land	5
Lw = 5	

^a^ Main land uses observed in Shiraz Plain

The final outputs are ranged from 28 to 280 and are classified according to Table 3.

**Table 3:** Vulnerability ranges corresponding to the CD index [9] and the NV index [12]

Vulnerability	Ranges (CD index)	Ranges (NV index)
Very low	<100	<70
Low	100–145	70–110
Moderate	145–190	110–150
High	190–235	150–190
Very high	≥235	≥190

### Nitrate vulnerability index (multiplicative model)

NV is another adaptation of the DRASTIC index, which was developed to achieve greater accuracy in estimation of the specific vulnerability to nitrate pollution. NV index is based on the real impact of each land use. This model attempts to integrate the risks of groundwater pollution by nitrate considering the land use as a potential source of nitrogen. The model incorporates potential negative and protective impacts uses that do not contribute to significant quantities of nitrate and do not enhance leaching of land uses overtime, such as the protected natural areas. It is based on a multiplicative model, involving the addition of a new parameter called the “potential risk associated with land use” (LU), which is calculated according to the following equation:

3 $$ NV\kern0.5em  index=\left(Dr\kern0.5em Dw+Rr\kern0.5em Rw+Ar\kern0.5em  Aw+Sr\kern0.5em Sw+Tr\kern0.5em Tw+\kern0.5em Ir\kern0.5em Iw+Cr\kern0.5em Cw\right). LU $$

Where LU refers to the potential risk associated with land use (according to Table 4) and the rest of the parameters are the same as equation 1.

**Table 4:** Ranges and ratings applied to the potential risk associated with land use (LU) as a source of nitrate pollution for the NV index [12]

Range	LU
Irrigated field crops	1.0
Urban areas	0.8
Uncultivated land and	0.3
Semi-natural areas	
Forests and natural areas	0.2

The final outputs are classified according to Table 3.

### Preparation of the needed layers

#### Depth to water table (D)

It is the depth from the ground surface to the water table. To get this layer, the most recent data regarding water level of 31 wells existing in the area of Shiraz plain was used. The interpolation method was applied to change the mentioned point data into raster map of water level. Finally, depth layer was prepared and was classified according to Table 1.

#### Net Recharge (R)

Is the amount of surface water that infiltrates to the ground and reaches the groundwater level. This study utilizes the Piscopo method [23] to prepare the net recharge layer for the Shiraz Plain based on the Table 5 and equation (4), below:

**Table 5:** Ranges and ratings applied to the Net Recharge parameter according to the Piscopo method [23]

Slope		Rainfall		Soil permeability		Recharge value	
Slope %	Factor	Rainfall (mm/year)	Factor	Range	Factor	Range	Rating
<2	4	850<	4	High	5	11–13	10
2–10	3	700–850	3	Moderate to high	4	9–11	8
10–33	2	500–700	2	Moderate	3	7–9	5
>33	1	500>	1	Low	2	5–7	3
				Very low	1	3–5	1

4 $$ Recharge\kern0.5em  index= Soil\kern0.5em  permeability+ Rainfall+ Slope\kern0.5em \left(\%\right) $$

In equation (4), the slope (%) was extracted from a Digital Elevation Model (DEM), which was generated from the topographic map of the Shiraz Plain and the Shiraz Plain soil map, log observations and exploration wells were used to quantify the soil permeability.

#### Aquifer media (A)

Is an indicator of material characteristics in the saturate zone. In this study, information from 20 well logs from Fars Regional Water Organization (FRWO) was used to prepare the aquifer media layer.

#### Soil media (S)

Is the top portion of the unsaturated zones that extends to the plants’ roots and organic creatures activity areas.

Soil map was prepared by using the soil map of Shiraz plain and log of observation and exploration wells.

#### Topography (T)

Is an indicator of land slope changes in the area. The abovementioned soil layer was used in preparation of this layer. The Topography Layer was prepared using the same method that was used in provision of net recharge layer and was classified based on the Aller table.

#### Impact of vadose zone (I)

Vadose zone is a layer in between the aquifer and the soil zone. The vadose zone characteristics show the attenuation behavior of the materials that are located above the groundwater table and below soil. This study used the lithologic data of 20 observation and exploration wells to develop the vadose zone media of the Shiraz Plain. Then, using this information and Table 1, we designed the raster map of Shiraz plain.

#### Hydraulic conductivity (C)

Is a property of an aquifer that describes the ability of water to move through the aquifer. We used 23 pumping tests data conducted by FRWO to generate the hydraulic conductivity layer. Table 1 was employed to rate the generated hydraulic conductivity layer.

#### Land use

This layer is imperative since it is required by both CD and NV indices. The 2009 IRS (Indian Remote sensing Satellite) data was used to generate this layer and then Table 2 and 3 were applied to rate it for preparing land use (L) map and land use associated risk (Lu), which are required for CD and NV indiced, respectively.

### Artificial neural network method (ANN)

In this study, the artificial neural network method (ANN) was used to present a model with higher performance and improve the DRASTIC method. For this purpose, input and output data (vulnerability) of DRASTIC model and the relevant nitrate values were divided into two categories of Train and Test. Vulnerability index values which were the results of drastic model, were corrected by nitrate values and model train was done by these corrected values. To conduct the test phase of the model, drastic parameters in the data of this phase were considered as input and groundwater vulnerability index was assumed as model output and the results were evaluated using nitrate concentration. Artificial neural networks are a mass information processing system that are parallel and have functions like human brain neural network [24]. The following principles are the basis of artificial neural networks: (1) Data processing takes place in individual units called neurons. (2) Signals between neurons are transmitted through communication lines. (3) The weight is assigned to communication line of that line. (4) Each neuron typically has activation functions and convertor to determine output signals from input data of the network [25]. Artificial neural network structure is in traduced by the pattern of connections between neurons, the method of determining the weights of communication and transfer function [26]. Normal structure of an artificial neural network is usually formed by the input layer, middle (hidden) layer and the output layer. The input layer is a transport layer and a mean for supplying the data. The last layer or the output layer includes predicted values by the network and therefore introduces the model output. Middle or hidden layers that are composed by processing neurons, are the place for data processing. The number of hidden layers and neurons in each hidden layer is usually determined by trial and error method. Neurons in adjacent layers in the network are fully linked together. Artificial neural networks are classified in various ways, such as how neurons are connected and data movement in the network [25]. In this study, the Multilayer Perceptron network which is one of the leading networks, was used where the information move input to the output. Neurons in one layer are not connected, but the neurons in one layer are connected to the neurons in the next layer. So the output of a neuron in a layer depends on the signal received from the previous layer, the weight assigned and the type of convertor function. Different steps in a network, are conducted by various mathematical algorithms in which the most important ones are: 1- BP: Back Propagation Algorithm, 2- CG: Conjugate Gradient Algorithm, 3- LM: Levenberg-Marquardt. Among which LM algorithm is the most efficient algorithm [27]. LM algorithm was used in this study.

### Nitrate Pollution Map

Groundwater nitrate concentration, as the most common pollutant in the study aquifer, can determine which of the used indexes offers greater precision in predicting the vulnerable areas. It is obvious that the model showing a higher correlation with the nitrate map will be more efficient. 82 groundwater samples were collected from 41 wells in a biannual sampling event, which took place in the months of August and January. The collected samples were analyzed for their nitrate concentration. The nitrate concentration distribution map was prepared using the average nitrate measurement in each point by employing ArcGIS 9.3 for interpolation.

### Statistical Analysis

A regression analysis was performed on the measured nitrate concentrations using the SPSS (Statistical Package for Social Science) statistical software (v. 19.0) to compare the three vulnerability indexes and to evaluate the consistency of each index with respect to the spatial distribution of nitrate pollution.

## Results and Discussion

### Depth to water table (D)

**Fig. 2:**
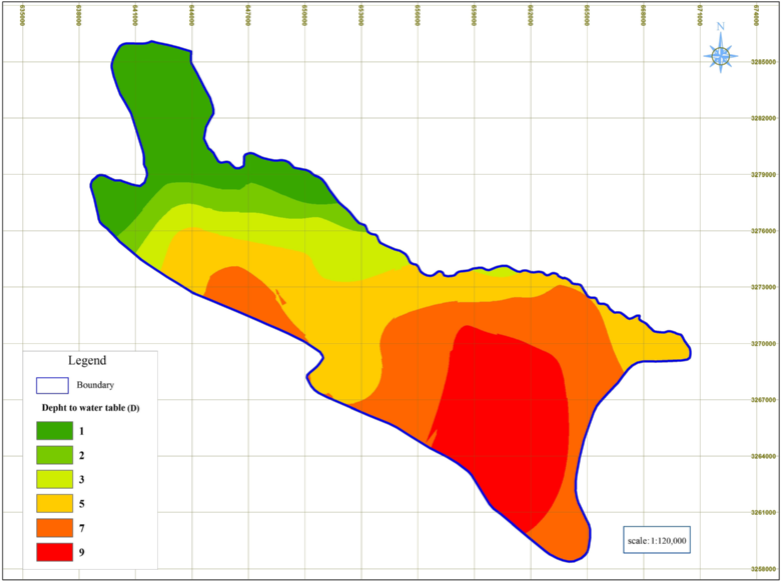
Rated maps of Depth to water table

Figure 2 and the DRASTIC model parameters rating table show that the groundwater table in the Shiraz Plain is from few meters to approximately 55 m. The results also indicate that the least effect of groundwater depth on groundwater vulnerability occurs in the northwest of the Shiraz Plain; whereas, the most effect of groundwater depth on vulnerability occurs in the south east part of the Shiraz Plain.

### Net recharge (R)

**Fig. 3:**
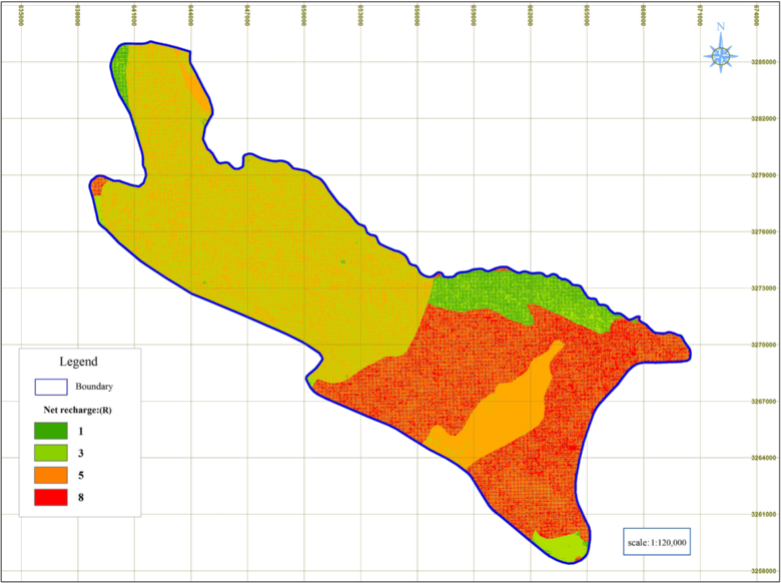
Rated maps of net recharge

Based on the Pscopo’s method the Shiraz Plain was divided in four (4) categories with respect to the net recharge, where the highest net recharge values were observed in southern and southeastern parts of the Shiraz Plain, where, the least net recharge values were observed in the north parts of the Shiraz Plain (Fig. 3).

### Aquifer media (A)

**Fig. 4:**
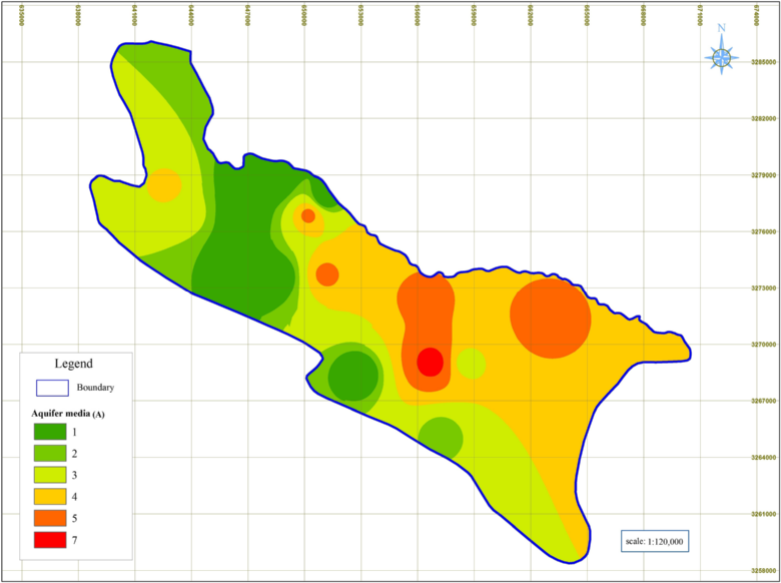
Rated maps of aquifer media

Figure 4 shows that majority of the Shiraz Plain is composed of clay and sit, where the coarse deposits can be found around Kaftrak Mountain.

### Soil media(S)

**Fig. 5:**
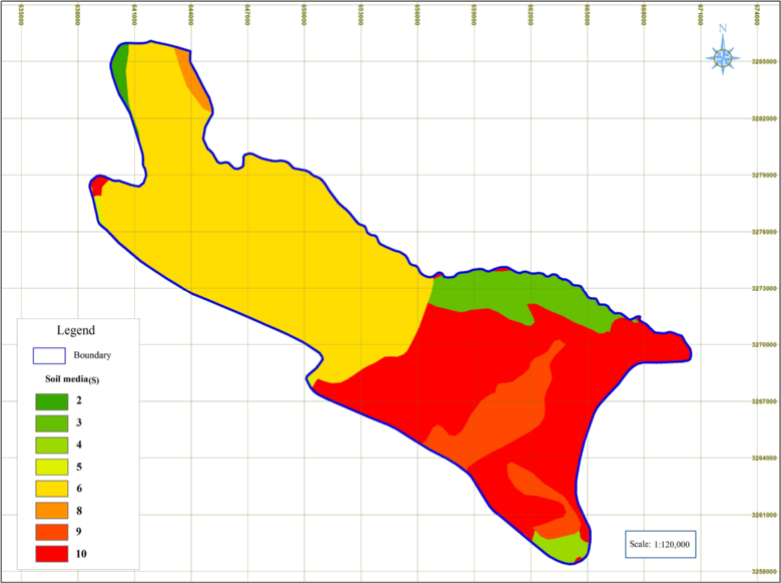
Rated maps of soil media

Figure 5 shows that an area from northwest to the center of the plain are sandy loam, where, moving to the southeastern parts, the thickness of the soil layer decreases.

### Topography (T)

**Fig. 6:**
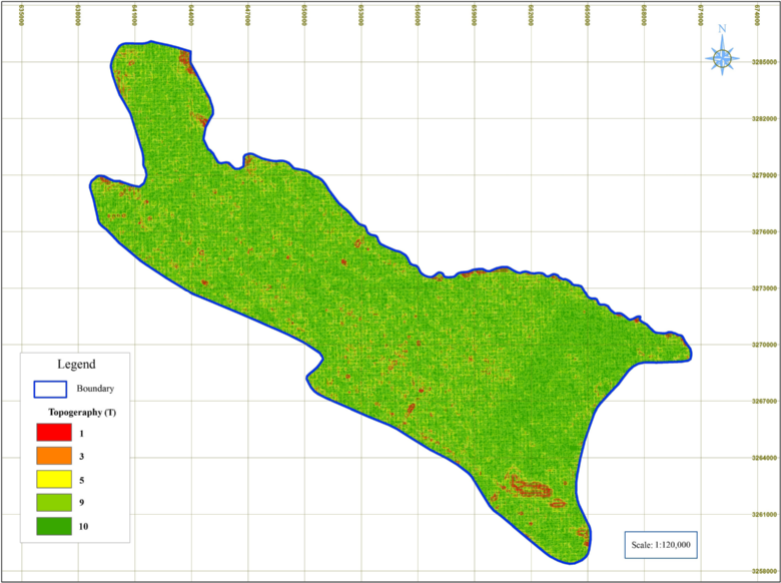
Rated maps of Topography

Figure 6 indicates that the Shiraz Plain slope varies from 0 to 2 % with the worst possible rating.

### Impact of vadose zone (I)

**Fig. 7:**
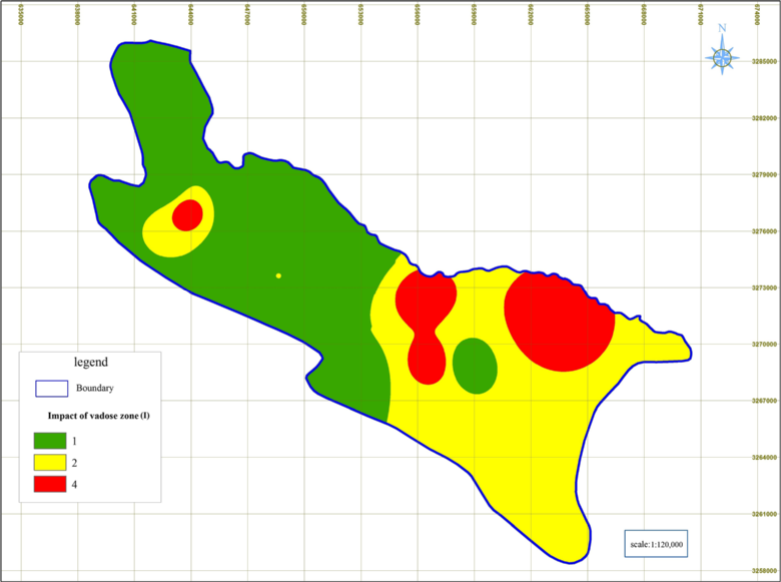
Rated maps of Impact of vadose zone

The results show that clay deposits exist in the north west toward southeast of the Shiraz Plain, where coarser media is seen in north and northeastern parts of the Shiraz Plain (see Fig. 7). The results also indicate that 87 % of the Shiraz Plain aquifer received rating values of one (1) to two (2).

### Hydraulic conductivity (C)

**Fig. 8:**
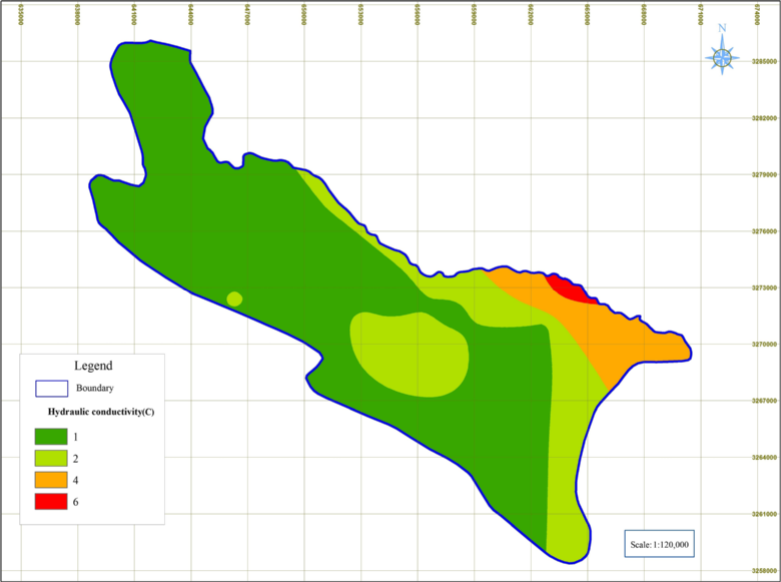
Rated maps of Hydraulic conductivity

The hydraulic conductivity of the Shiraz Plain aquifer was found to be varied from 0.34 m/day to 37 m/day, where majority of the aquifer have the hydraulic conductivity of around 12 m/day (Fig. 8) and high conductivity parts are in the east of the aquifer.

### Land Uses (L) and Potential risk associated with land use (LU)

**Fig. 9:**
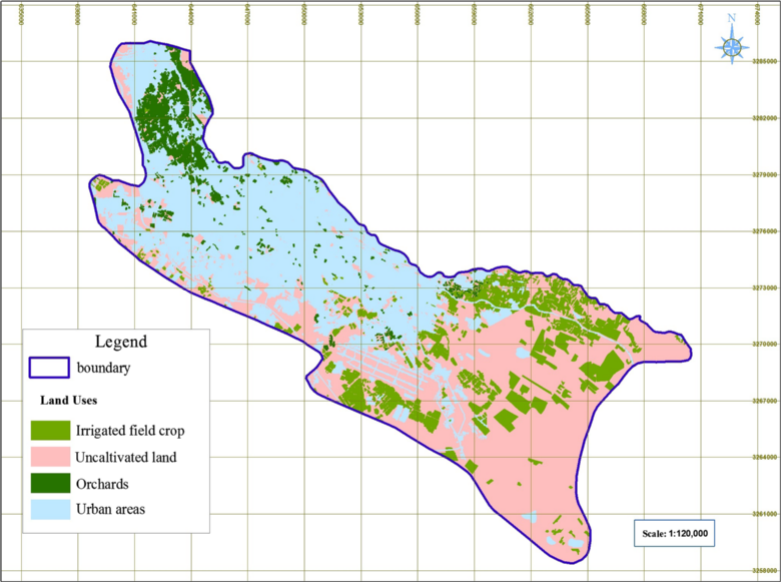
The major land uses classes in the study area

**Fig. 10:**
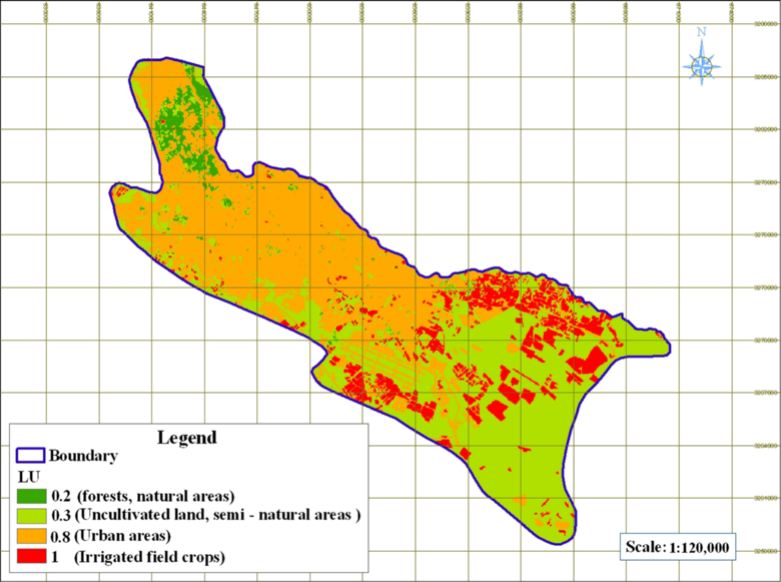
Rated map of potential risk associated with land use (LU)

The results in Figs. 9 and 10 show that portions of uncultivated land, urban areas, agricultural lands, and orchards are 45.67, 38.29, 11.32, and 4.72 % respectively.

#### Specific vulnerability of groundwater to nitrate pollution according to the CD index

**Fig. 11:**
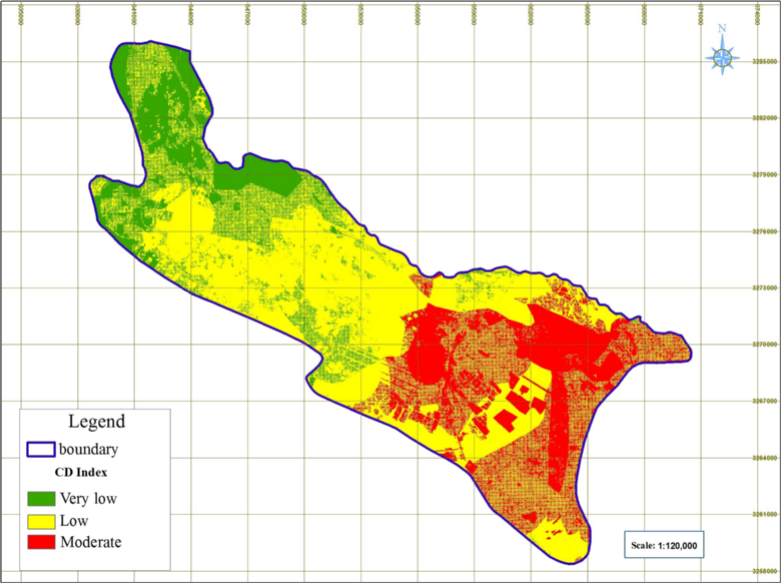
Map of specific vulnerability to nitrate pollution according to the CD index

The final CD index was calculated using equation 2 through multiplying the rated layers by their weights and integrating them in GIS (Geographic Information System). Then, zoning of Shiraz plain’s vulnerability map was done (Fig. 11). Accordingly, CD index for Shiraz aquifer varied from 53 (very low) to 185 (medium) and was divided into three classes as follows: 19 % of the area; i.e., northern and northwestern parts, had very low risk of pollution, 25 % of the area, mainly southeastern areas, showed moderate vulnerability to nitrate pollution, and other parts of the aquifer (56 %) were at low risk.

#### Specific vulnerability of groundwater to nitrate pollution according to the NV index

**Fig. 12:**
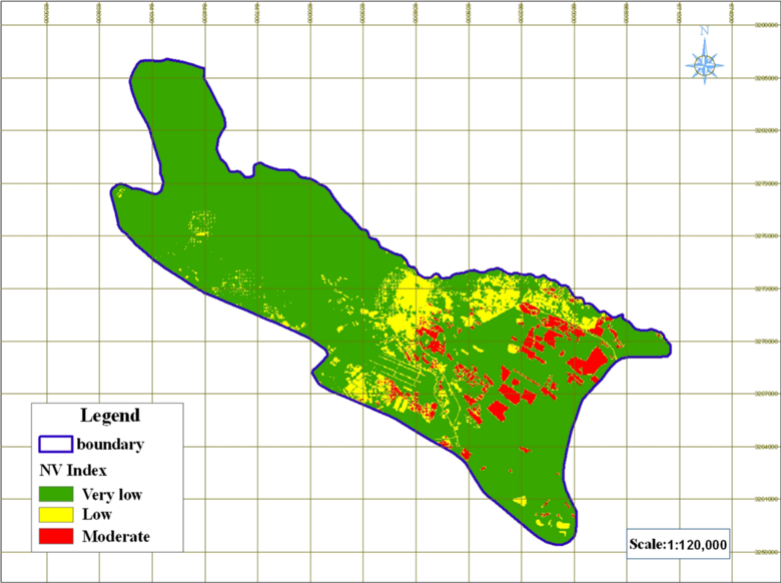
Map of specific vulnerability to nitrate pollution according to the NV index

The results for specific vulnerability to nitrate pollution according to the NV index have been presented in Fig. 12. The map demonstrated that the NV index of Shiraz plain ranged from 6.4 to 185, and was divided into three classes: very low (<70), low (70–110), and medium (110–145). On this basis, 6.45 % of the study area located at central and southeastern parts of the plain had moderate vulnerability, whereas the remaining 81.9 % were of very low and low vulnerability. There were no areas in the high and very high vulnerability classes.

#### Artificial neural network method (ANN)

**Fig. 13:**
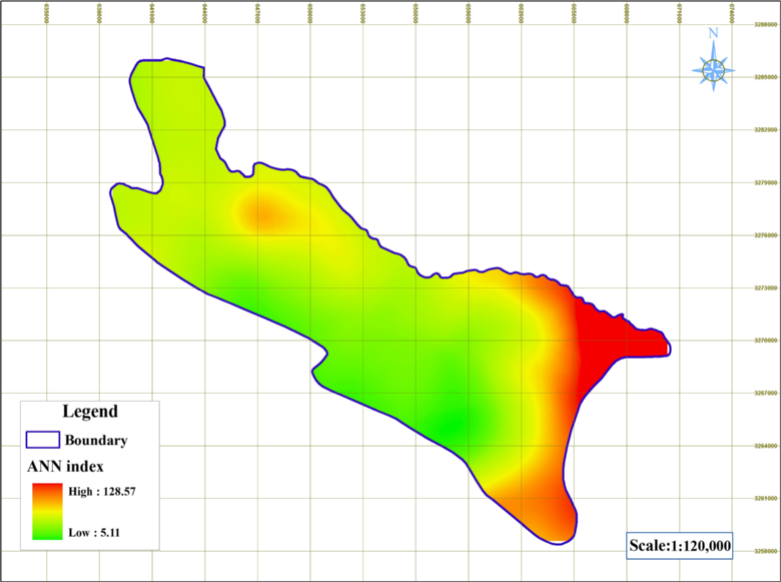
The vulnerability map using ANN method

In order to predict the vulnerability of groundwater by artificial neural networks method, perception perceptron three-layer network with LM algorithm was used. In this method, 7 input parameters including drastic parameters were used as the input layer. The number of neurons in the middle and output layers is 9 and 1, respectively. LM algorithm was used to train the network; the details of training and the calculation process is provided by the American Society of Civil Engineers [25]. The convertor transfer function is in the second layer of the tangential sigmoid (tansig) and the third linear layer (purlin). The number of courses epoch was 100 and the coefficient of determination and RMSE (Root Mean Square Error) values were 0.90 and 7.55, respectively. After training, the model was conducted for test phase step and vulnerability coefficient predicted by nitrate values was obtained. In Fig. 13, vulnerability map is provided by artificial neural network. As seen in the figure, majority of the region has low vulnerability and the regions adjacent to Maharloo Lake in the South East part of the region and the northern part of the plain have more vulnerability. Comparing this map with the map of nitrate, it can be seen that there is a good conformity between nitrate distribution in the region and vulnerability. Nitrate is increased in the south part of the region and areas adjacent to Maharloo Lake, as well as some parts in the North; and according to ANN map, vulnerability in these areas is higher than other estimated areas.

#### Comparison of CD, NV and ANN models

**Fig. 14:**
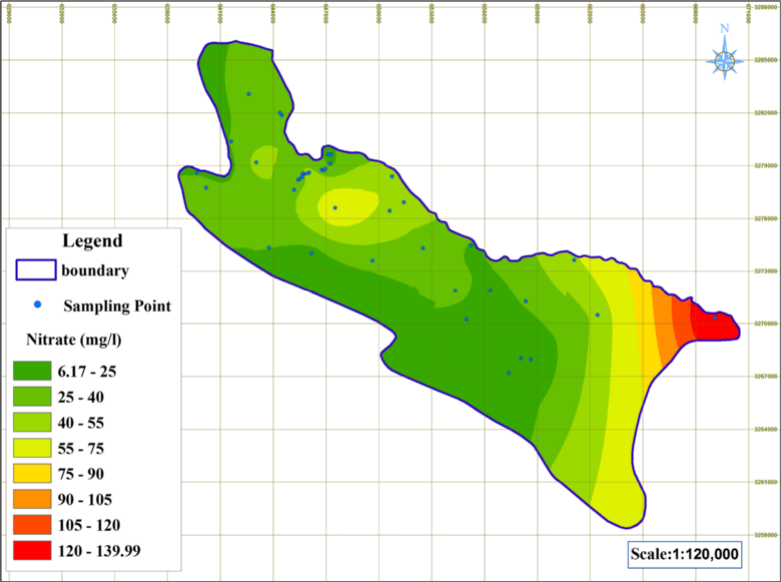
Map of nitrate pollution of groundwater in Shiraz aquifer

The groundwater nitrate pollution map for Shiraz aquifer is presented in Fig. 14. Based on this results the southeast part of the study area has these highest nitrate concentration and the western part of the study area has the lowest nitrate concentration. This confirms the results of NV, CD and ANN models, since the areas with higher concentration of nitrate have higher risk in comparison to the areas with lower risk. The vulnerability maps based on these three indexes showed similar results, identifying the southeastern part of the aquifer as the vulnerable zone. However, the percentage of the areas in the moderate class in the NV index was lower compared to that in the CD index. The results of regression analysis indicated a significant quadratic non-linear relationship between groundwater nitrate concentration in the study area and NV and CD and ANN model values (*P* < 0.01). Moreover, the ANN models showed better r-squared value and greater accuracy compared to the NV and CD indexes regarding nitrate distribution (Table 6).

**Table 6:** Regression analysis results using the mean nitrate concentration of groundwater in the Shiraz aquifer

Model	R Square
CD	0.292
NV	0.303
ANN	0.80

The results showed that the ANN model was the most effective model to improve the DRASTIC index, compared with CD and NV indexes. This can be justified by three reasons: (1) ANN as a nonlinear model is able to better explain nonlinear behavior of aquifer that is a complex system, (2) In contrast to NV and CD methods, supervised training method adopted corrected vulnerability to train the ANN model, (3) The ANN model reduces subjectivity of model by using LM optimization method to reach to suitable DRASTIC model. In addition according to our findings, the NV index allowed improved accuracy in determination of the vulnerable areas and showed better correlation coefficient compared to the CD index. This result was consistent with the observations of Martinez-Bastida J et al. [12] who proposed the NV model for the first time and concluded that their new type of multiplicative model offered greater accuracy. With respect to their viewpoint, this is due to the fact that the NV index incorporates both the negative impacts of some land uses over time and also the protective effects that others may have upon the aquifer media (uses that do not contribute to significant quantities of nitrate and do not enhance leaching, such as the protected natural areas. Nevertheless, it seems that the real reason for higher accurate of the NV index is that in this index, maximum rating (1) is assigned to irrigated field crops and lower rating (0.8) is allocated to urban areas. In the CD index, on the other hand, a same rating (8) is assigned to both agricultural and urban areas, but construction of sewerage systems in the recent years has caused leakage of nitrate from urban uses to decrease, so that in some regions, including Shiraz, this type of land use is not considered as a main nitrate source any more. As a result, it is not reasonable to assign the same rating to land uses with different effects. This seems to be the main reason why the NV index was more accurate in the present study.

As mentioned above, most parts of Shiraz aquifer had very low and low vulnerability. Although depth of water table in these parts was shallow, they contained fine-grained sediments which led to decrease of surface recharge and the possibility of increase in occurrence of attenuation process, including chemical degradation, absorption, and dispersion [28, 29]. This can be noticed from the rated maps of the aquifer media, vadose zone, and hydraulic conductivity (Figs. 4, 7 and 8). On the other hand, NV, CD, and ANN models determined the southeastern parts of the study area as the most vulnerable areas. This can be explained by the shallow groundwater depth of aquifer, low thickness of the saturated area, lack of soil layer, the general slope of the Shiraz Plain, which is toward this area, and the high volume of agricultural activities in the area. These facts imply that the traditional irrigation methods, such as flood irrigation, result in low N-use efficiency with high risks to the southeastern part of the study aquifer. The impact of irrigation on enhanced nitrate leaching has also been reported by some other researchers [30–33]. In one study, it was found that before irrigation, NO_3_ concentration was less than 20 mg/L, while irrigation accompanied by fertilizer application caused NO_3_ concentration in the upper layers of the aquifer to reach 65 mg/L [34]. It is necessary to say that nitrate leaching can be reduced through: (i) the use of crops with high N-use efficiency during the cropping period, (ii) a fertilization strategy aimed at synchronizing fertilizer application to meet crops demand through split applications or the use of slow release nitrate-N fertilizers, (iii) an effective irrigation and water management system to minimize water losses and increase water use efficiency by the crops, (iv) an integrated approach using a good water and fertilizer management system and crops with high N-use efficiency, and (v) avoiding intensive agriculture in vulnerable areas where nitrate leaching potential is high.

Another main reason for the higher nitrate concentrations in the southeastern parts of the plain was elevated groundwater level. In order to overcome this problem, three drainage lines are being constructed in southern and southeastern parts of Shiraz plain during the recent years. However, the efficiency of the drainage system in this area decreases due to the fine grain soil in the area near Maharlu Lake and at the end of the drainage.

## Conclusion

In this study, we assessed the vulnerability of Shiraz unconfined aquifer using CD (based on additive formulation), NV (based on multiplicative formulation), and ANN (based on artificial intelligence) models. The results confirmed the usefulness of these models for evaluating the risk of nitrate pollution in Shiraz plain. However, the map of specific vulnerability based on the ANN model proved to be more accurate with respect to the real nitrate distribution in groundwater within the study area. Based on the findings, more than two-thirds of Shiraz aquifer had very low and low vulnerability. Three models confirmed that areas with high volume of agricultural activities and shallow groundwater depth were the most vulnerable zones for nitrate contamination. Based on these results, optimized irrigation techniques and lower consumption of fertilizers are suggested for the vulnerable zones. The results of our study could serve as a scientific basis in future for sustainable land use planning and groundwater management in Shiraz plain.

## Abbreviations

AI, Artificial Intelligence; ANN, Artificial Neural Networks; BP, Back Propagation Algorithm; CD index, Composite DRASTIC index; CG, Conjugate Gradient Algorithm; DEM, Digital Elevation Model; FRWO, Fars Regional Water Organization; GIS, Geographic Information System; IRS, Indian Remote sensing Satellite; LM, Levenberg-Marquardt; MLP, Multi-Layer Perceptron; NV index, Nitrate vulnerability index; RMSE, Root Mean Square Error; SPSS, Statistical Package for Social Science; USEPA, United States Environmental Protection Agency.
